# Towards tailored and targeted adherence assessment to optimise asthma management

**DOI:** 10.1038/npjpcrm.2015.46

**Published:** 2015-07-16

**Authors:** Job FM van Boven, Jaap CA Trappenburg, Thys van der Molen, Niels H Chavannes

**Affiliations:** 1 Unit of PharmacoEpidemiology & PharmacoEconomics, Department of Pharmacy, University of Groningen, Groningen, The Netherlands; 2 Department of Rehabilitation, Nursing Science & Sports, University Medical Center Utrecht, Utrecht, The Netherlands; 3 Department of General Practice, University Medical Center Groningen, University of Groningen, Groningen, The Netherlands; 4 Department of Public Health and Primary Care, Leiden University Medical Center, Leiden, The Netherlands

## Abstract

In this paper, we aim to emphasise the need for a more comprehensive and tailored approach to manage the broad nature of non-adherence, to personalise current asthma management. Although currently several methods are available to measure the extent of asthma patients’ adherence, the vast majority do not incorporate confirmation of the actual inhalation, dose and inhalation technique. Moreover, most current measures lack detailed information on the individual consequences of non-adherence and on when and how to take action if non-adherence is identified. Notably, one has to realise there are several forms of non-adherence (erratic non-adherence, intelligent non-adherence and unwitting non-adherence), each requiring a different approach. To improve asthma management, more accurate methods are needed that integrate measures of non-adherence, asthma disease control and patient preferences. Integrating information from the latest inhaler devices and patient-reported outcomes using mobile monitoring- and feedback systems (‘mHealth’) is considered a promising strategy, but requires careful implementation. Key issues to be considered before large-scale implementation include patient preferences, large heterogeneity in patient and disease characteristics, economic consequences, and long-term persistence with new digital technologies.

## Introduction

A key driver for asthma treatment success is the extent to which patients adhere to their prescribed pharmacological regimen.^1^ Good adherence encompasses multiple dimensions, including intensity and timing of use according to prescription (compliance), continuous use (persistence) and correct use (inhalation technique).^2,3^ Non-adherence to asthma medication is associated with poor clinical, humanistic and economic outcomes.^4–6^ Previous studies showed that adherence to asthma medications on average is far from optimal. Depending on methods, population and setting, estimates of non-adherence typically range between 30 and 75% in both adults and children.^7,8^ Current methods to measure medication adherence include direct biochemical measurement, clinician judgment, self-report, assessment of prescription refill data and the use of electronic devices.^8,9^ These methods all have their advantages and disadvantages. However, an important limitation that most methods share is that they do not incorporate confirmation of actual inhalation, dose and inhalation technique. Furthermore, most methods fail to provide detailed information on the individual consequences of non-adherence and on when and how to take action if non-adherence is identified. Therefore, more accurate methods are required that integrate measures of the broad nature of non-adherence and essential elements of asthma control. Combining patient-reported outcomes with the latest inhalation-, adherence monitoring- and feedback systems offers a promising new strategy.

The aim of this paper is to assess the value of current asthma adherence measures in relation to the management of diverse types of non-adherence, levels of asthma control and patient preferences and engagement. Subsequently, a more comprehensive non-adherence-managing approach is proposed and discussed.

## Identification of papers

To identify relevant papers, a semi-structured search in Pubmed and Cochrane was performed with several combinations of the following search terms: adherence, asthma, inhalers, mobile phone and electronic monitoring. Papers deemed relevant were studied, and additional papers were retrieved through searching the references.

## The broad nature of asthma non-adherence

In the management of asthma, good understanding of the diversity and complexity of patient adherence is required.^2^ As such, the World Health Organization has defined three types of non-adherence: erratic non-adherence, intelligent non-adherence and unwitting non-adherence.^2^

Currently, the terms intentional and unintentional non-adherence are also commonly used.^10^

### Erratic non-adherence

Erratic non-adherence is probably the most well-known type of non-adherence. This form of unintentional non-adherence is often referred to as forgetfulness.^2^ Patients have the intention to be as adherent as possible, but simply cannot combine the medication regimen with their busy daily life.

### Intelligent non-adherence

A patient with ‘intelligent’, or intentional, non-adherence, purposely alters, discontinues, or even fails to initiate prescribed therapy. This deliberate non-adherence reflects a reasoned choice, albeit not necessarily a wise one.^2^ Patients who ‘feel’ better may decide that they no longer need to take their medications, i.e., their illness perception has an important role.^11^ A number of factors may be responsible: fear of perceived short- or long-term side effects; taste; complexity; interference with daily life; cost; disagreement with provider regarding need. All these factors may cause patients to choose to avoid daily therapy.^2,11^

### Unwitting non-adherence

Another form of unintentional non-adherence is unwitting non-adherence. Unwitting non-adherence is the failure to understand fully either the specifics of the regimen or the necessity for adherence.^2^ Unwitting non-adherent patients make no intentional decision to be non-adherent. Studies have repeatedly confirmed that patients frequently forget instructions given to them during a clinic visit.^12^ In asthma management, it is common for patients to misunderstand the difference between ‘as needed’ medication and maintenance medication. Another example of common ‘unwitting non-adherence’ is poor inhaler technique.^3,13,14^

## Managing non-adherence in asthma: not simply one-size fits-all

Most of the erratic non-adherent patients could be helped by straightforward interventions such as simplification of the dosing regimen, electronic reminders, or the advice to link the intake of medication to a daily habit.^2^ In contrast, patients that are intelligent non-adherent do not need any reminders, but may be more likely to benefit from a process of shared decision making and motivational interviewing.^15^ Finally, unwitting non-adherent patients are expected to benefit from extra education.^9^ For this purpose, self-management and written action plans have been widely recommended in international and many national guidelines.^16,17^ The effectiveness of these plans has been proven;^18^ however, uptake of written action plans is still suboptimal.^19,20^ In addition, there is room for improvement of the specific elements impacting effectiveness of these plans, especially regarding suitability in patients with limited literacy.^21^ Patients with poor inhalation technique would be helped by more information and training to enhance their skills regarding inhaler use. Thereby it should be mentioned that training and education on inhalation technique have to be provided by well-trained healthcare professionals. Notably, some studies suggest that there is ample room for improvement regarding medical personnel’s knowledge on inhalation devices.^22,23^

## Detecting specific types of non-adherence in asthma: current limitations

To aid the healthcare provider in detecting and assessing asthma patients’ adherence, several methods are available. Most commonly used methods include direct biochemical measurement (e.g., in blood or other body fluids), judgment of healthcare providers, patient self-report, assessment of prescription refill data, and the use of electronic monitoring devices.^8,9^ In general, the specific value of the current measurement methods regarding detecting and managing erratic non-adherence, intelligent non-adherence and unwitting non-adherence has been under-reported, but is considered a key issue for optimal management and outcomes. For example, erratic non-adherence often remains unnoticed until exacerbations occur.^5^ Intelligent non-adherence may be detected during patient interviews with a healthcare provider, given that the patient is willing to share his concerns.^10^ However, the topic is often only covered substantially in the consultation room once it is too late (e.g., after severe exacerbations). Another possibility to detect intelligent non-adherence is by the use of validated self-report instruments such as the MARS-A questionnaire.^24^ Yet, asking the patient might result in a ‘socially acceptable answer’ and thereby an overestimation of actual adherence.^25,26^ Unwitting non-adherence, e.g., a poor inhalation technique, might be diagnosed by a healthcare provider observing the patient, but usually there would need to be another reason for consultation first. Although guidelines highlight the importance of regular inhalation technique assessment as part of every asthma review, real-life practice often falls short.^17,27^ Currently, patients themselves cannot rely on valid instruments to assess the quality of their inhalation.

The value of current methods regarding the three types of non-adherence and other aspects is compared and summarised in Table 1. For a more extensive comparison, we refer to previous reviews that compared adherence measurement methods in asthma.^8,9,28^ Table 1 was the result of thorough assessment of these previous reviews and a final consensus between the authors of this article.

Direct biochemical measurement is an unbiased method to assess actual intake, but is costly and invasive and only provides a point estimate of adherence.^9,28^ In-depth patient interviews by a healthcare provider can provide insight in most types of non-adherent behaviour, yet they require regular face-to-face meetings and effective patient–provider communication to obtain a continuous measure.^10,29^ Longitudinal measurement of adherence by prescription refills provides a continuous and remotely accessible alternative.^28^ However, this method of measuring adherence lacks discriminative properties regarding the three types of non-adherence and cannot detect a poor inhalation technique.

In general, most methods routinely used fail to take into account the interaction between different forms of non-adherence and variations in asthma control, thereby wasting the opportunities for tailored, proactive interventions. In conclusion, each of the current methods available has its strengths, but none of the methods are optimal for tailored asthma non-adherence management.

## Optimising adherence assessment in asthma: requirements

**Figure 1:**
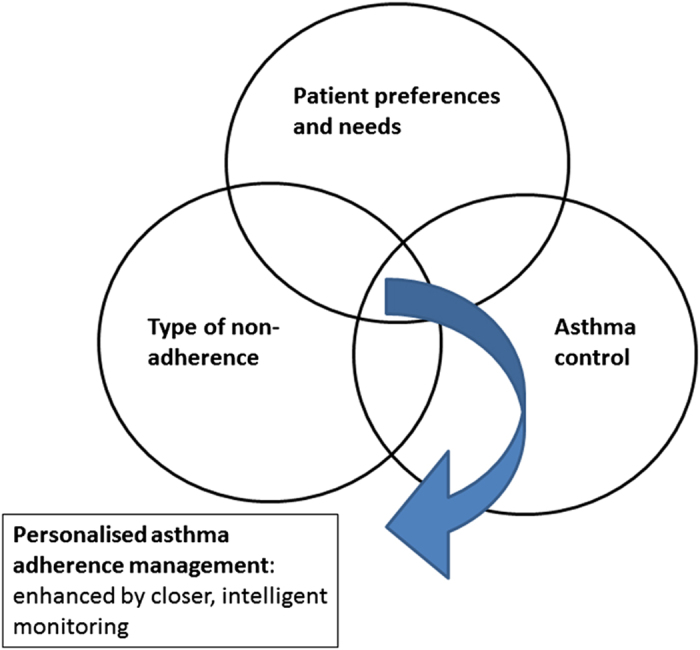
Personalised asthma adherence management by taking into account three domains.

Without disregarding the value of a good initial diagnosis and the wide availability of a basic level of asthma care, the limitations described in the previous section demonstrate the need for an integrated, personal, approach to deal with the broad nature of asthma non-adherence. This approach needs to take into account not only the type of non-adherence, but also patient preferences and asthma control (Figure 1). In our view, this requires the digital integration of the latest inhalation technology and asthma control monitoring tools in a patient-preference-based environment.

### Detection of non-adherence

Ideally, non-adherence detection aids will provide us with both a qualitative measure of adherence (e.g., inhaler handling and inhalation technique) and a continuous, quantitative measure to detect all types of non-adherence. The quantitative measure includes the frequency, hour of the day and time interval between inhalations. Electronic monitoring devices, attached or integrated in inhalation devices, appear the most promising to capture both aspects.^30^ These electronic devices have been recommended as the reference standard to assess medication adherence in both research and real-world clinical settings.^31^ Technically, these devices have shown their feasibility and some have been available on the healthcare market for almost 20 years, although they are still primarily used in small-scale research settings.^32^ Examples of monitoring devices that are able to log the date and time of actuations are the SmartMist, Doser CT, MDIlog and the Nebulizer Chronolog, of which studies demonstrated that all devices were sufficiently accurate.^33,34^ A more recent development is the Propeller Health sensor that not only keeps a record of time and date, but also of location of use.^35^ Another development is the Smartinhaler that automatically sends usage data to a mobile application or computer via Bluetooth and can provide discreet audio-visual reminders if the patient forgets to take the prescribed medication.^36^ Yet, all devices still do not assess the quality of inhalation. A promising new technique relies on the use of acoustic inhalation measurements. In research settings, time-stamped acoustic recordings seem to be a suitable method for monitoring inhalation technique over time.^32,37^ Interestingly, an association between better adherence and changes in asthma quality of life and peak expiratory flow was only found when adherence was operationalised as both time of use and inhalation technique, and not for time of use only.^32^ These developments illustrate that some of the technology to introduce an electronic monitoring component within asthma management is already available.

### Asthma control

Two of the most frequently used health status tools to measure asthma control are the Asthma Control Questionnaire (ACQ-6) and the Asthma Control Test.^38,39^ In current clinical practice, these tools are only occasionally used, most often during regular patient consultations. Interventions following from these asthma control scores are generally reactive. In contrast, continuous digital monitoring of asthma control would enable early detection of increasing symptoms and proactive interventions. Theoretically, whenever ACQ/Asthma Control Test scores exceed a certain threshold (based on previous scores and preferences), alerts and decision support could be sent to the patient and/or healthcare provider. Subsequently, initial remote assessment of the patient (e-consultations) could be performed, only followed by active in-person consultation if deemed relevant. In stable periods, no consultations would be needed, even when non-adherence is signalled. First results from studies assessing e-consultations showed that patients were satisfied with an e-consultation process,^40^ but research is still very limited and no specific studies in asthma have been performed. Therefore, more studies are obviously necessary before large-scale implementation should take place.

### Patient preferences

The asthma population is very heterogeneous regarding its needs, preferences and capabilities.^41^ For example, patients with low health literacy and self-management ability may need a different approach, especially as these aspects are associated with worsened outcomes.^42,43^ Also patients’ current use and preferences towards mobile phone-based management may have a role in both uptake and effectiveness of this digital strategy. In renal transplant recipients, 79% showed a positive attitude towards mobile phone-based adherence and health monitoring, but their attitude was positively influenced by smartphone ownership.^44^ In asthma, disease severity and comorbidities may influence patients’ preferences and abilities for more or less self-care.^45^ A cross-sectional study indicated that about 60% of the asthma patients preferred an active or collaborative role in treatment decisions and 40% a passive role, regardless of disease severity.^46^ As regards this heterogeneity, a three-point approach to enhance patients’ motivation and to optimise outcomes has been suggested.^47^ This approach includes counselling on the necessity of asthma medication, addressing patients’ concerns about medication, and tailoring the choice of the treatment regimen to patients’ preferences and capacities.

In addition, the possibility for asthma patients to interact with their peer patients may be beneficial. Peer-to-peer contact has been shown to improve self-efficacy and asthma control with favourable cost-effectiveness; however, studies are limited.^48–50^ Finally, acceptance of digital asthma health care, in particular, depends on age, duration of disease, education and use of computers/Internet.^51^

## The concept of integrating adherence measurement, asthma control and patient preferences

Developments in inhalation technology and digital asthma control measurement are promising. These technologies offer the potential to enhance adherence and tailor asthma management, that could result in improved quality of life and reduced healthcare utilisation.^3,4,52,53^ However, to achieve optimal intervention uptake and effect size, careful conceptualisation of the design is essential. Ideally, adherence measurements of electronic inhalation devices are directly transferred to an mHealth platform on a mobile phone. First, this will provide patients with real-time insight in their own medication-taking behaviour, but second, these data can be extracted during regular consultations with their healthcare providers. It is unlikely that the healthcare provider will be monitoring these signals on a daily basis, but the server could be set up to issue alarms, should control fall outside of certain preset levels. The information gathered would help in the performance of the next structured asthma review. In this context, such a system could fall under M for Monitoring in SIMPLES or under S for Support.^54^ Integrating information regarding the timing and quality of inhalations with patient-reported outcomes has the potential to provide the full picture; that is, it may be able to signal patterns regarding forms of non-adherence and subsequent poor asthma control. On the basis of these observations, personalised treatment and recommendations can be provided, depending on patients’ needs. For some patients, automatically generated, but tailored, feedback by decision-aid systems may be sufficient. For other patients, however, direct in-person and digital consultations are required for optimal patient-centred care, education and outcomes. In addition, the digital platform will allow patients to interact with their peers. It may also serve as a place where healthcare providers can provide general treatment and lifestyle recommendations, given that patient confidentiality is always taken into account.

**Figure 2:**
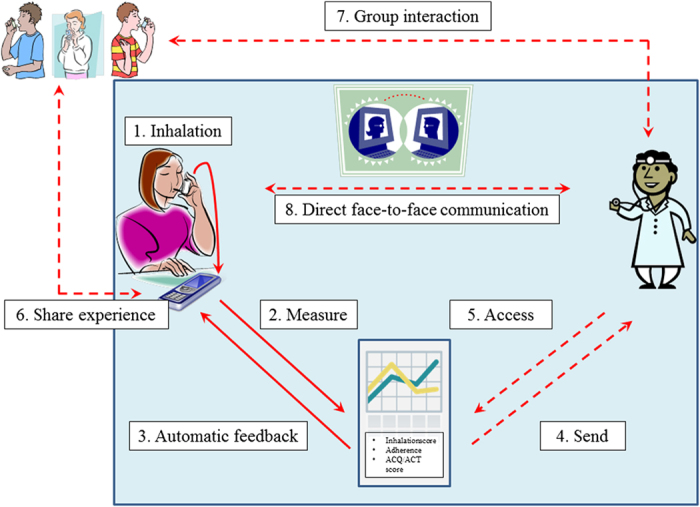
Integrated asthma adherence management approach. Solid red lines: standard mHealth decision support; dashed red lines: optional pathway, depending on patient and healthcare provider preferences and feasibility studies.

A global outline of this approach is visualised in Figure 2. It includes the use of a smartinhaler combined with a mobile phone application to measure adherence and asthma control, tailored decision support and motivational feedback, and optional digital or direct face-to-face contacts with healthcare providers and/or peer patients. As depicted, several opportunities for tailored asthma management are offered, depending on patients’ and healthcare providers’ needs and preferences (more self-management or more guidance by healthcare providers) and feasibility studies.

## Optimising adherence assessment in asthma: considerations

This integrated approach seems promising and fits well with the current movement towards personalised, predictive, preventive and participatory respiratory medicine (‘P4 medicine’).^55^ Yet, an important barrier to innovation is that technologies such as these are often considered to be too costly.^56^ Regarding the innovative technology itself, it is expected that product costs will rapidly decrease over time.^30^ Less predictable, however, are the economic consequences of this potential shift from primarily ‘scheduled’ chronic care to future ‘on-demand’ care. It is likely that face-to-face consultations would reduce as the patients learnt better how to control their disease.^57^ It is, however, unclear whether this would also lead to fewer hospitalisations. Therefore, careful analysis of current chronic digital asthma care is necessary. A review by Huckvale *et al.* ^58^ systematically assessed the content of 103 asthma apps and compared the contents to international guidelines and best practices. The authors concluded that none of the different apps combined reliable information about asthma with supportive tools for self-management. A recent Cochrane review reported on the effectiveness and cost-effectiveness of digital apps for asthma self-management and found that the current evidence is not sufficient to advise large-scale implementation.^59^

Long-term adherence to these technologies is one of the other issues that should be taken into account. A study from 2004 suggested that patients used an asthma-monitoring website only for a limited amount of time.^60^ Note that this study was performed in times before the smartphone era. A more recent study, by van Gaalen *et al.*,^61^ did indeed show persistence of effect in the intervention group. Evidence of the economic dimension of more comprehensive digital asthma management tools suggest neutral to unfavourable cost-effectiveness, but evidence is limited and highly dependent on implementation and current technology costs.^57,62^

The question rises whether this technology should be restricted to selected populations. Should we for instance focus on the mild asthma population, in whom fewer in-person contacts may be safe, or should we focus on the persistent asthma population, where disease burden and costs are highest? Another possibility is that these technologies become an integrated part of the medication, just as is currently the case with existing inhaler devices. Manufacturers would not only provide the pharmacological component, but also the technology to assist patients and healthcare providers in optimising asthma disease management. Especially in the current era of limited major pharmacological breakthroughs but a wealth of new inhalers available, the provision of these kinds of services may significantly increase the added value of manufacturers and make them stand out from the rest. Other issues that need to be addressed include privacy issues, data ownership, and digital competences of patients and healthcare providers.

In general, the most successful telemonitoring implementations have occurred when all of the basics (including sample size, intervention, duration of follow-up and definition of outcomes) have been done properly and monitoring is integrated into a system that is ready to receive it.^63^ However, these systems are much less likely to have a positive impact unless all the other prerequisites are in place, which is why so many telemonitoring trials fail to demonstrate benefit. When used as part of an integrated pathway,^64^ mHealth support has the opportunity to improve asthma outcomes, but more research regarding optimal implementation is highly required.

## Conclusion

Current measures to assess asthma non-adherence do not provide sufficient information. More targeted and personalised methods, combining measures of non-adherence, asthma control and patient preferences are needed. Mobile monitoring systems offer a promising integrated approach to assess (non)-adherence and optimise asthma management, but require careful implementation.

## Figures and Tables

**Table 1:** Characteristics of current asthma medication adherence measurement tools

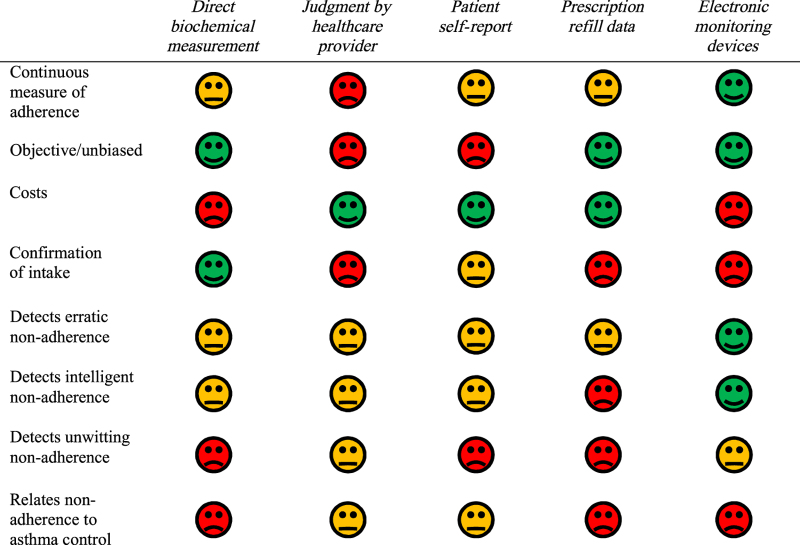

Green: positive; orange: medium; red: negative.

## Notes

NHC is an Associate Editor of *npj Primary Care Respiratory Medicine*, but was not involved in the editorial review of, nor the decision to publish, this article. The other authors declare no conflict of interest.
